# Bioethical considerations at times of Zika virus

**DOI:** 10.1590/S1679-45082016ED3725

**Published:** 2016

**Authors:** Marco Aurélio Scarpinella Bueno, Henrique Grunspun

**Affiliations:** 1 Hospital Israelita Albert Einstein, São Paulo, SP, Brazil.

Last March, many experts were brought together in Geneva in a meeting organized by the World Health Organization (WHO) to discuss the dramatically increase of Zika virus infection in the Americas.^(1)^ Among attendees there were virologists, immunologists, neurologists and epidemiologists – all of them concerned on how to stop the rapid progress of Zika virus in Brazil and in other countries in the Americas.

The Zika virus, in the past an agent responsible for occasional disease in humans, is today responsible for outbreaks in Brazil and Latin America of microcephaly, Guillain-Barré syndrome, myelitis and meningoencephalitis cases,^(2,3)^ in such a degree that WHO declared a public health emergency situation of international concern.

However, the particular increase of microcephaly cases^(3-7)^ related to maternal infection by Zika virus that works as an excellent model for discussion of a number of bioethical issues, such as the dilemma of healthcare professionals to assist pregnant woman who will have a baby with microcephaly, the autonomy principles of mother and fetus, the discussion on authorization of abortion in cases of fetal malformation (an idea posed by the Non-Governmental Organization – ANIS – responsible for the lawsuit issued in 2012 that authorized, after approval of Brazilian Supreme Court, pregnancy termination for anencephalic fetuses), patient-physician relationship models, and resources replacement and equality in access to health system.

In bioethics, there is no absolute and immutable truth. However, there is a number of concepts and a set of actions that aim to identify and help to solve a number of ethical dilemmas appearing daily in relation to patient care.

For this reason, we consider the context itself (the ethical dilemma) and its aggravating and relieving factors before take a decision that, sometimes, is not ideal, but for a specific case, seems to be more adequate.

Bioethics approach requires a continuous behavior both in medical practice and in behavior as citizens. Sometimes such roles are overlap such as in the case of women who inquiry physicians about Zika virus infection. This situation to discuss only about delay pregnancy, sometimes so expected by the woman, however, when the pregnancy occurs, there is the need to discuss issues related with risk of have a baby with microcephaly.

Today, it seems to be easy to discuss with women to not become pregnant. Especially due to the uncontrolled outbreak and news that appear every day, this seems a clever behavior, but we cannot deny that we are interfering in the free will of future mothers.

Obstetricians and infectious disease specialists are able to assess statistical risk of fetal malformation appearance that is generally considered remote and occasional, and they do not usually influence patient’s decision to become pregnant. However, what about the risk of microcephaly?

Other cases of microcephaly associated with other factors are well known and continue to occur. Such cases are similar or more prevalent than Zika virus. These factors are alcoholism, poor controlled gestational diabetes and other perinatal infections as toxoplasmosis, rubella, cytomegalovirus and syphilis.

However, since November 2015, temporal relationship between Zika outbreak and alarming increase of microcephaly prevalence has indicated a strong relationship among these events. Confirmation of virus presence in the amniotic fluid of two pregnant women from Paraiba State (Brazil),^(4,5)^ whom had symptoms compatible to Zika virus and gave birth of microcephaly babies. The virus identification in the tissue sample of children who born with microcephaly^(6)^ lead the Brazilian Ministry of Health to confirm the relationship between the virus and microcephaly,^(7)^ a fact that has been causing concern to pregnant women.

Officially, the Brazilian Ministry of Health reinforces pregnant women to perform qualified prenatal test, and prescribed tests, and also they must report their physician any clinical changes, in addition to continue with preventive measures against *Aedes aegypti* (use of long sleeves, repellent, mosquito net, and elimination of standing water in and around home). Pregnant women should consult their physician before travel to risk areas.

The Brazilian Ministry of Health recommends to health professionals careful evaluation of cephalic perimeter and gestational age during prenatal, and also notification of suspected cases of microcephaly at birth. Because health professionals are always in contact with population, they must reinforce measures to combat the mosquitoes spreading the virus.

However, in the age of Dr. Google consult, facts can become more controversial. The Centers for Disease Control (CDC) in newsletter published last March 11, recommended pregnant women to not travel for areas where Zika virus transmission is occurring,^(8)^ a similar position was stated by WHO directors.

More controversial than cancel a travel is discuss if women living in areas with Zika outbreak must postpone pregnant, which sometimes was intensively planned. One scenario is to delay pregnancy at 25-years-old, a different one is request this delay for women older than 40 years (it is important to remember that a recent resolution of Brazilian Federal Medical Council authorized woman older than 50 years to perform artificial insemination treatment without previous authorization).

Who can guarantee that this outbreak will pass quickly? Recently, the CDC issued guidance recommending men and women diagnosed with Zika to wait before decided to have a child. According to CDC, women must wait for at least 8 months after beginning of symptoms to become pregnant. For men the recommendation is to wait at least 6 months.^(9)^

In case of exposition to risk areas where Zika virus circulates recommendation to try conception is 8 weeks. Such guidance are based on limiting data available about virus permanence in the blood and semen. To date, there is no evidence that fetus conceived after disappearance of virus in the bloodstream is at risk to develop the infection.^(9)^

Some Brazilians’ experts remember that such recommendations of CDC applies to American context, where no outbreak has occurred yet, and they can be used as safety measures for persons diagnosed with Zika from now on. An agreement among Brazilian scientists is that CDC decisions about the time to become pregnant are personal, complex and must be individualized case by case, and also based on guidance of an obstetrician.

Data analysis of literature indicates that number of microcephaly notifications in children who born from mothers infected with Zika virus in Brazil has increased more than 20 times if compared to notification from previous years.^(2,4)^

Since the beginning of investigations by the Brazilian Ministry of Health in October 2015, a total of 7,150 notifications were recorded until April 16, 2016. Of the cases already finished, 2,241 were excluded and 1,168 microcephaly cases and other nervous system changes suggesting congenital infection were confirmed.^(10)^ A total of 3,741 cases are still under investigation.

Of 1,168 cases, 192 have been already confirmed in laboratory tests for Zika virus. However, the Brazilian Ministry of Health emphasizes that such data do not represent adequately the total number of cases related to the virus, considering that there was infection by Zika virus in the majority of mothers who had babies with microcephaly.^(10)^

Until April 16, 246 fetal or neonatal deaths were recorded (3.4% of notified cases) with suspicion of microcephaly and/or central nervous system change after delivery or during pregnancy (abortion or stillbirth). In 51 cases, microcephaly was confirmed. Others 165 cases continue under investigation, 30 were excluded.^(10)^

It is know that most of pregnant women exposed to Zika virus will give birth to babies without microcephaly, but the real risk of occurrence of malformation still generating controversial information even in the medical literature.

Data analysis of Zika virus outbreak reached the French Polynesia between 2013 and 2014 suggesting risk of occurrence of microcephaly in about 1% of cases in which the infection occurred in the first trimester of pregnancy,^(11)^ (*i.e.*, less than the risk of microcephaly associated with other congenital infections such as cytomegalovirus and rubeolla).

Kleber de Oliveira et al.^(6)^ reported prevalence of microcephaly significantly higher (2.8 *versus* 0.6 cases of microcephaly per 10,000.00 birth alive) in the Brazilian States where active transmission of Zika virus occurs.^(6)^ A recent cohort study^(12)^ carried out by group from Rio de Janeiro, Brazil, included 88 pregnant women with at least 5 days of pruriginous skin rash. In 72 women (82%) the infection by Zika virus was confirmed and it might occurred between 5th and 38th weeks of pregnancy.

The most concern aspect of this study was that 29% of obstetric ultrasound performed in 42 pregnancies with positive test for Zika (58%) show very significant abnormalities as delayed intrauterine growth with or without microcephaly, ventricular calcifications, oligohydramnios and arterial flow changes, and two fetal deaths.^(12)^

Although medical community have advanced in little period of time concerning Zika virus infection, studies lack to answer a variety of questions, such as vertical transmission rate and data on infected fetuses who show complications.^(13,14)^

In April 2016, CDC, based on rigorous review of scientific evidence, confirmed the relationship of Zika virus and occurrence of microcephaly, attributing to the virus the brain damages identified in fetuses.^(3)^

As a matter of fact, all this insecurity of having a child with microcephaly have relight the controversial debate about women right of control over their body and, as a consequence, to abort.

How to deal with birth of a child with severe microcephaly? What is the future impact of this sequela? Are there conditions for full inclusion of this child in the society? Does the mother have the right to abort?

This mother, under strong emotional impact and, sometimes, abandoned by her partner, is in condition to decide autonomously?

The principle related with autonomy constitutes one of the four fundamental premises of bioethical proposed by Beauchamp et al.,^(15)^ in addition to beneficent of nonmaleficence and justice.

However, we can clearly understand the bioethical point of view over such complex subject, and also the different approaches it involves. Other important author on bioethics, Levinas,^(16)^ proposed an alterity model, (*i.e.*, “putting oneself in the other’s place”, according to definition of Professor José Roberto Goldim.^(17)^ Therefore, it means to talk about values).^(18)^

Lévinas^(16)^ invertes the golden rule “treat others the same way you want them to treat you”. The others discoveries are what impose the adequate conduct. When the golden rule “Your rights end where mine begin” does not make sense anymore, what become to be valid is “my rights are guaranteed by others rights”.^(17)^

In this context of individual values of each patient, many times different from our own values, pregnant women’s opinion must be always respected, because some of them, due to their believes, are contrary to interrupt the pregnancy, other pregnant women with different views must have their option respected as well.

As a support for bioethical decision-making is recommended the deep assessment and knowledge about ethical and legal behavior that support the physician, specially: code of medical ethics that guides the fundamental principles of medicine, including physicians rights, professional responsibility, relationship with patient and families, among other topics; the Brazilian civil rights law that regulates legal relationship between individuals in several levels, the Criminal code that defines what is considered crime and which responsibilities each citizen has.

It is never too late to remember how devastating are the consequences of microcephaly over underserved population. Not only for lack of access of children to needed therapies on the future (physical rehabilitation, speech-language and hearing therapy, occupational therapy) but also because of terminate of unintended pregnancy in unfavorable socioeconomic conditions is often related to high rates of maternal morbidity and mortality.

What about the young, single and poor girls living in risk areas who do not know and/or cannot prevent unintended pregnancies, particularly do not know how to plan such pregnancies and end up becoming victims of their pregnancy, of *Aedes aegypti*, Zika virys and microcephaly of their babies?

As stated by Professor Segre^(19)^ “the internal conflict of a healthcare professional facing a terminal disease, a mother willing to abort, or maintenance of confidentiality in risk situations that involve others, is part of him/her, and immune to the law.”
